# Senescent Macrophages and the Lung Cancer Microenvironment: A New Perspective on Tumor Immune Evasion

**DOI:** 10.14336/AD.2024.1404

**Published:** 2025-12-27

**Authors:** Lexin Qin, Tingting Liang, Xinyu Zhu, Wentao Hu, Bo Li, Meidan Wei, Jiaxin Zhang, Jianxiang Li, Jin Wang

**Affiliations:** School of Public Health, Suzhou Medical College of Soochow University, Suzhou, Jiangsu, 215123, China.

**Keywords:** Tumor microenvironment (TME), macrophage senescence, immunosenescence, senescence-associated secretory phenotype (SASP), lung cancer

## Abstract

Lung cancer treatment is evolving, and the role of senescent macrophages in tumor immune evasion has become a key focus. This study explores how senescent macrophages interact with lung cancer cells, contributing to tumor progression and immune dysfunction. As aging impairs macrophage functions, including phagocytosis and metabolic signaling, it promotes chronic inflammation and cancer development. p16^INK4a^-positive macrophages are common in aged mice, and their clearance slows tumor growth, suggesting these cells support tumor proliferation and immune evasion. Targeting the senescence-associated secretory phenotype (SASP) and reprogramming senescent macrophages offers potential therapeutic benefits, including reversing immune aging and boosting anti-tumor immunity. However, translating these findings into clinical practice requires further molecular understanding and rigorous clinical trials.

## 1. Introduction

The tumor microenvironment (TME), characterized by its diverse components and dual functionality plays a crucial role in the development and progression of lung cancer [1, 2]. Macrophages, which are abundant immune cells within the TME, influence tumor growth, invasion, and metastasis and participate in various aspects of immune surveillance [3, 4]. In healthy tissues, macrophages, as essential elements of the host defense system, can recognize and eliminate abnormal cells, including early-stage tumor cells and senescent cells [5-7]. However, in the TME, macrophages often become subverted by advanced tumor cells, losing their original anti-tumor functions [8, 9]. Macrophages are primarily classified into two types: M1 macrophages, which counteract tumors by producing pro-inflammatory cytokines and cytotoxins, and M2 macrophages, which support tumor growth by secreting growth factors and pro-angiogenic factors [8, 10]. The balance of M1/M2 macrophage polarization determines the fate of organs during inflammation or injury. Recent studies have shown that macrophages activated in certain diseases exhibit a senescent phenotype and promote disease progression [11, 12]. Therefore, macrophage senescence is associated with various diseases.

Cellular senescence is a complex biological process characterized by cell cycle arrest, DNA damage, oxidative stress, resistance to apoptosis, accumulation of senescence-associated β-galactosidase (SA-β-gal), and senescence-associated heterochromatin foci [13]. Senescence can result from telomere shortening due to repeated cell divisions, which activate the DNA damage response pathway and subsequently the tumor suppressor p53, known as replicative senescence [14, 15]. Additionally, cellular stress, such as oxidative stress, ionizing radiation, or cytotoxic treatments, can activate the cell cycle inhibitor p16^INK4a^, known as stress-induced senescence [15]. These pathways may interact, ultimately activating the cell cycle inhibitor p21^CIP1^, leading to cell cycle arrest by inhibiting cyclin-dependent kinases [16]. Thus, senescence can be considered a defense mechanism against cancer [17, 18]. However, the accumulation of senescence-associated secretory phenotype (SASP) products promotes tumor development, indicating that senescent cells play a role in tumorigenesis which is dependent on the environment. The dual role of senescence in tumor suppression and promotion remains unclear and is influenced by the cellular context and the composition of the SASP [19-23]. The accumulation of senescent macrophages has gained increasing attention in aging and age-related diseases, including cancer [24-26]. Owing to their functional decline, these cells trigger a series of adverse effects within the TME, promoting lung cancer cell proliferation, immune evasion, and therapeutic resistance [26, 27].

Recognizing the role of senescent macrophages in the lung cancer microenvironment provides novel therapeutic targets. Strategies targeting these cells could enhance lung cancer therapies, particularly as immunotherapy becomes a standard treatment for lung cancer. Therefore, understanding the interactions between senescent macrophages and lung cancer cells, and their mechanisms in tumor immune evasion is not only scientifically innovative but also clinically significant.

## 2. Immunosenescence

As observed in various physiological responses, the immune system undergoes characteristic functional and phenotypic deterioration with age, leading to weakened responses to pathogens and cancer cells and promoting the development of age-related chronic inflammation, such as neurodegenerative diseases and cancer. These changes are collectively described as "immunosenescence" [27-31].

### 2.1 Causes of immunosenescence

Several factors significantly influence this process, including genetics, sex, exercise, nutrition, history of microbial exposure, and human cytomegalovirus infection [32-35]. A prominent phenotype of immunosenescence is the gradual involution and degeneration of the thymus with age, resulting in decreased T cell output [36, 37]. Additionally, age-related inflammation induces a senescence-associated secretory phenotype (SASP), further contributing to immune system aging [38, 39]. Thymic involution significantly impacts the proportional balance of T cells in the immune system [40, 41]. The thymus comprises epithelial tissue and peri-vascular spaces devoid of thymopoietic function. With aging, the thymus progressively atrophies, the epithelial regions shrink, and the peri-vascular spaces expand, occupying the senescent thymus. This process leads to a reduction in naïve T cells, an increase in peripherally late-differentiated memory T cells, and a diminished capacity for naïve T cell migration to the periphery [42-44]. Studies indicate that young individuals who undergo thymectomy due to congenital heart disease exhibit early immunosenescence, with T cell characteristics similar to those of elderly individuals, underscoring the role of thymic involution in immune system aging [45].

### 2.2 Characteristics of immunosenescence

A key feature of immunosenescence is "inflammaging," a chronic low-grade inflammatory state characterized by elevated inflammatory markers in the blood, which is considered a core factor of aging [46]. This process is triggered by the accumulation of damaged molecules and endogenous cellular debris, leading to sustained tissue damage [47]. Cellular senescence plays a crucial role in this process, with *in vivo* studies showing the gradual accumulation of senescent CD8+ cells, as observed by Effros RB *et al*. [48]. Senescent T and B cells contribute to elevated pro-inflammatory cytokines, increased reactive oxygen species (ROS), and dysfunctional lysosomal deposition, potentially exacerbating autoimmune diseases and increasing the risk of various illnesses [12, 49, 50]. These findings suggest a close association between the underlying mechanisms of tumorigenesis and immunosenescence. Furthermore, decreased lymphopoiesis and reduced adaptive immunity lead to increased myelopoiesis, resulting in a several-fold increase in macrophages in certain organs [51, 52].

As the immune system ages, metabolic changes, including enhanced glycolysis, impaired mitochondrial function, and increased ROS, also occur [53, 54]. These metabolic alterations are highly associated with various age-related diseases common in elderly individuals, such as cardiovascular diseases, neurodegenerative diseases, autoimmune diseases, metabolic disorders, and cancer [55]. With the increasing incidence of these diseases in the elderly population, shared cellular and molecular mechanisms may drive their progression [17]. Exploring these molecular processes, changes in immune cell populations, and their signaling regulation is crucial for understanding the link between immunosenescence and age-related diseases.

## 3. Macrophage senescence

Immunosenescence has profound impacts on individuals, particularly in aging populations, and the understanding of cellular senescence in immune cells, especially innate immune cells such as macrophages, is only beginning to unfold [56, 57]. Senescence may disrupt the physiological gene regulation and functions of macrophages, such as reducing phagocytic capacity, altering autophagic and metabolic signaling, and impairing inflammatory responses, among others. Re-establishing the immune balance of macrophages could potentially reverse some aspects of senescence, which may have therapeutic potential in aging and age-related diseases [11, 58-60]. Macrophages are present in almost all tissues of the body, play critical roles in both nonspecific and specific immune responses, and facilitate the maintenance of tissue homeostasis as well as in tissue repair and remodeling.

### 3.1 Inducers of macrophage senescence

Macrophage senescence can be induced by multiple internal and external factors, which are detailed below. These factors may trigger changes in gene expression, cell cycle regulation, and functional decline.

(a) Senescence-Associated Secretory Phenotype (SASP): Hall *et al*. demonstrated that the SASP of senescent cells could induce a senescence-like phenotype in young mouse macrophages, characterized by increased expression of the tumor suppressor p16^INK4a^ and β-galactosidase (β-gal) activity, both of which are considered reliable markers of senescence in *in vitro* and *in vivo* models [61].
(b) Pathogen Exposure: *Pseudomonas aeruginosa* PAO1 infection has been shown to induce macrophage senescence through the NF-κB signaling pathway [62, 63]. Likewise, lipopolysaccharide (LPS), a well-known macrophage activator, promotes senescence by activating NF-κB, leading to increased BRD4 expression [64].
(c) Ionizing Radiation: Exposure to ionizing radiation (e.g., 10 Gy) can elevate p16^INK4a^ expression and activate SASP in lung macrophages, contributing to impaired immune function and elevated p16 and SASP expression [65].
(d) Hypoxia: Similarly, under hypoxic conditions, macrophages exhibit senescence-like characteristics, including p21/p53-mediated cell cycle arrest, morphological changes, and enhanced SA-β-gal activity [66].
(e) Metabolic Factors: High glucose levels have been reported to induce senescence in both endothelial cells and macrophages *in vivo* and *in vitro*, promoting a SASP response [67].
(f) Genetic Factors: In a Kras-driven mouse model of lung cancer, Prieto et al. observed an early accumulation of senescent cells, particularly alveolar macrophages, characterized by increased expression of p16^INK4a^ and Cxcr1. Clearance of these senescent macrophages mitigated adenoma formation and tumor progression, suggesting that senescent alveolar macrophages may promote tumorigenesis [26].

These observations highlight the dual role of tissue-resident macrophages in supporting both tissue repair and tumor progression by altering their local microenvironment. The senescence of lung macrophages may promote the progression of various chronic lung diseases, a mechanism that requires further in-depth study.

### 3.2 General changes in macrophage senescence

**Figure 1.**
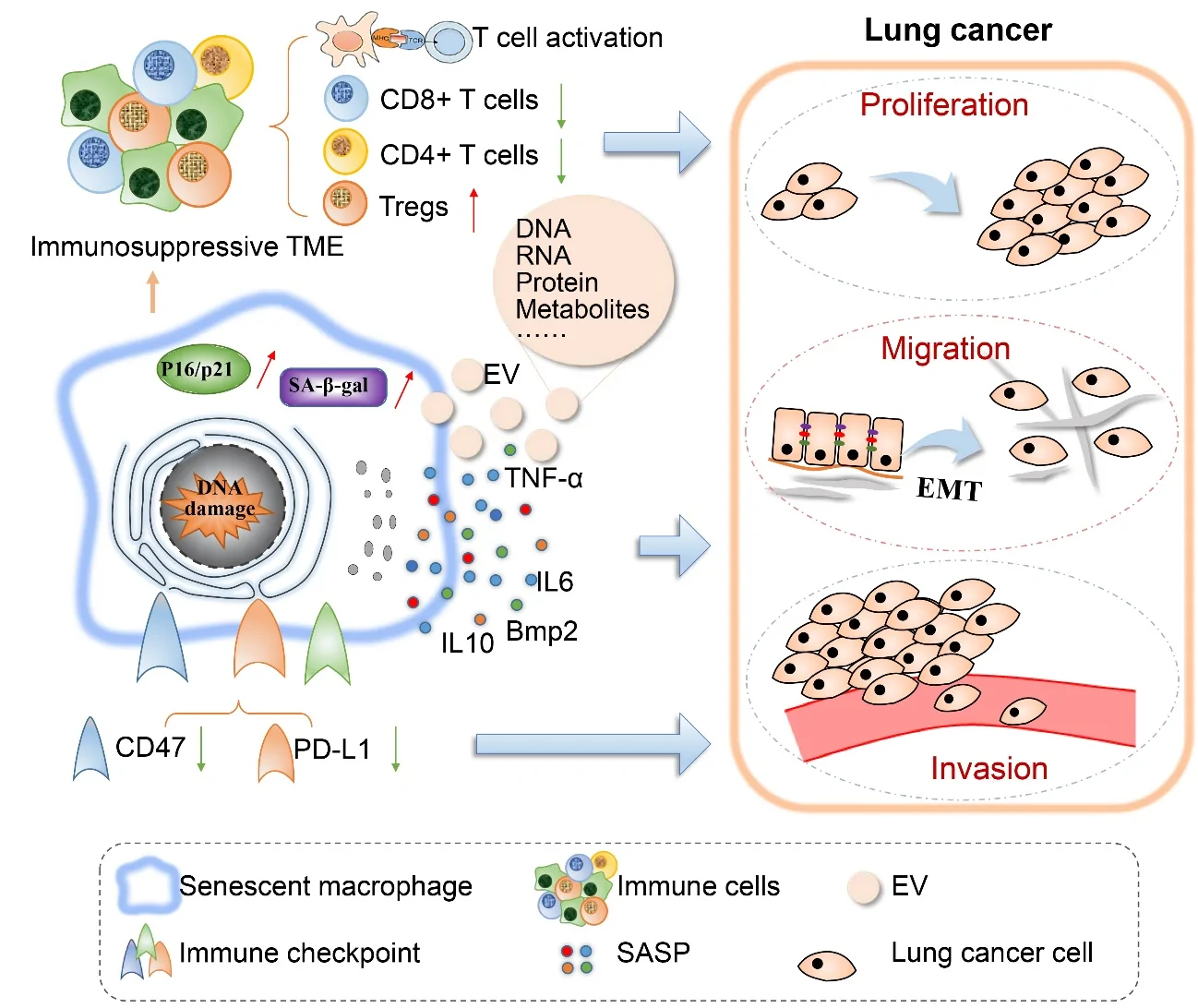
**The mechanisms by which aging macrophages promote lung cancer**. Treg, Regulatory T cells. TME, Tumor Microenvironment. SASP, Senescence-Associated Secretory Phenotype. EMT, Epithelial-Mesenchymal Transition. EV, Extracellular Vesicle.

Macrophage senescence is a cellular state closely associated with inflammation, immune responses, and tissue repair, characterized by a series of molecular markers [68]. Identifying and characterizing senescent macrophages *in vivo* remains challenging, due to the absence of unique and universally accepted markers for this state. Commonly used senescence assessment markers include cell cycle inhibitors, such as p16^INK4a^ and p21, whose elevated expression is considered indicative of cellular senescence. First, macrophage senescence exhibits changes that are similar to those seen in the senescence of other cell types (Fig. 1), as detailed below.

(a) SA-β-gal activity: Notably, while increased SA-β-gal is a hallmark of cellular senescence due to lysosomal compartment expansion in senescent cells, its use as a marker in macrophages remains controversial [69]. This is because monocyte differentiation into macrophages is associated with an increase in lysosome numbers [70], and lysosomal volume also increases following LPS stimulation to maintain cellular functionality [71]. Therefore, SA-β-gal activity may not always be a reliable marker of senescence in macrophages, requiring further validation in different contexts.
(b) Gene expression: Accumulation of p16^INK4a^-positive macrophages was observed in young mice induced by senescent cells [61]. Additionally, systemic clodronate treatment suggests that p16^INK4a^-positive macrophages constitute a majority of senescent cells in aged mice. Under ionizing radiation senescence-specific markers such as p16^INK4a^ are upregulated in lung macrophages [65, 72], leading to functional impairments such as defects in phagocytosis. *Pseudomonas aeruginosa* can drive macrophages toward senescence, increasing p16^INK4a^ levels [62].
(c) SASP and Cytokine Secretion: Senescent macrophages often exhibit an altered secretory profile known as the senescence-associated secretory phenotype (SASP). This secretory profile includes increased production of pro-inflammatory cytokines such as IL-6, TNF-α, and IL-1β, which play key roles in promoting chronic inflammation and are implicated in various age-related diseases, including cancer [73, 74]. At the same time, senescent macrophages may also secrete anti-inflammatory cytokines like IL-10 to counterbalance excessive inflammation [75-78]. Additionally, increased secretion of chemokines CCL2 (MCP-1) and CXCL8 (IL-8) may promote the recruitment of monocytes and other immune cells, exacerbating inflammatory responses [79].
(d) Reactive Oxygen Species (ROS): In addition to cytokine secretion, senescent macrophages often produce elevated levels of ROS, which can further impair cellular function and contribute to the aging process.

### 3.3 Specific changes in macrophage senescence

Specific markers and functional changes inassociated with macrophage senescence have been observed in various tissues (Fig. 1).

(a) CD68 and CD11b Expression: In normal aging, the macrophage-specific protein CD68 has been shown to increase in specific brain regions of male C57BL/6NNia mice [80]. Furthermore, CD11b (also known as integrin alpha M, ITGAM), which is involved in adhesion interactions between monocytes, macrophages, and granulocytes, is upregulated in senescent macrophages, suggesting functional alterations in the activated state [81].
(b) Glucose Metabolism: GLUT1, a key glucose transporter, has been implicated in promoting a pro-inflammatory phenotype in macrophages under high glucose conditions, a feature closely associated with cellular senescence [67, 82]. Inhibiting GLUT1 significantly reduces mTOR phosphorylation and SASP production in high glucose-induced senescent macrophages, indicating a potential therapeutic target to modulate senescence and inflammation in macrophages [82].

Molecular markers of macrophage senescence not only reflect their senescent state but are also closely related to their functions in immune response and inflammation. Understanding these markers is crucial for studying the roles of macrophages in aging, disease, and therapy.

## 4. Senescent macrophages promote lung cancer

Tumor-associated macrophages (TAMs) are among the most abundant and critical immune cells in the tumor microenvironment (TME) [83]. They play roles in angiogenesis, extracellular matrix remodeling, cancer cell proliferation, and metastasis [83]. However, macrophages are highly plastic cells and can exhibit different functions at various stages of tumor development [84]. Given their significant contribution to the TME, several studies have shown that macrophage senescence can also influence cancer progression (Fig. 1). Senescent macrophages may impact lung cancer progression through various mechanisms, including altered cytokine secretion and modulation of other immune cells (e.g., T cells), thereby reshaping the immune microenvironment. Importantly, these senescent macrophages may create an immunosuppressive milieu that facilitates tumor immune evasion, which is a critical challenge in lung cancer therapy [85].

### 4.1 SASP in senescent macrophages promotes lung tumor progression

Cellular senescence is associated with the secretion of a senescence-associated secretory phenotype (SASP), which includes factors that can stimulate tumor cell proliferation, migration, and invasion, thereby promoting tumor progression within the senescent microenvironment. SASP is rich in inflammatory cytokines and chemokines that contribute to "inflammaging" and tumor progression [86]. SASP factors can also directly regulate immune responses and impact tumor development [87]. Conditioned media from senescent fibroblasts have been shown to promote tumor cell growth, indicating that SASP can enhance tumor proliferation through paracrine signaling [88, 89]. Senescent macrophages promote tumor growth directly by producing heterogeneous SASP and fostering angiogenesis. Macrophages expressing p16^INK4a^ produce several oncogenic SASP factors uniquely present in tumorigenic lungs, including Bmp2, Ccl2, Ccl7, Ccl8, Ccl24, Cxcl13, IL-10, TNF-α, IL-6, and IL-1β [90]. BMP-2 is highly overexpressed and specifically localized in non-small cell lung cancer (NSCLC), where it promotes tumor cell growth, migration, and invasion [91]. TNF-α also favors tumor cell growth and invasion [92]. CCL2 facilitates cancer cell migration and recruits immunosuppressive cells to the TME via interaction with CCR2, aiding cancer progression [93]. IL-6 promotes the proliferation and differentiation of cancer stem cells (CSCs), enhancing tumor invasiveness and resistance [94]. IL-10 creates an immunosuppressive environment conducive to angiogenesis, proliferation, and metastasis [95]. These factors not only establish an immunosuppressive TME but also serve as critical mediators in promoting tumor growth and invasion through paracrine signaling.

### 4.2 Tumor-promoting effects of senescent macrophage-derived extracellular vesicles

In addition to soluble factors, senescent cells secrete abundant extracellular vesicles (EVs), which are also components of SASP [96-98]. EVs are critical players in intercellular communication, transporting various biomolecules and organelles [99-101]. Recent studies have indicated that EVs play significant roles in cellular senescence [102], aging [103], and age-related diseases [104, 105]. Senescent bone marrow monocytes/macrophages can undergo senescence to distant tissues via EVs, causing age-related dysfunctions [106]. Senescent macrophages secrete increased EVs containing ribosomal proteins, major vault proteins, pro-inflammatory miRNAs (e.g., miR-21a, miR-155, miR-132), and several mRNAs [107]. Moreover, EVs from senescent macrophages can enable senescent mouse embryonic fibroblasts to reinitiate cell proliferation, which may account for their tumor-promoting activity [107]. Haston's study revealed disruption of the tumor vasculature and significant tumor volume reduction upon senescent macrophage clearance, indicating that senescent macrophages may promote tumor growth by increasing tumor vascularization and thereby supplying sufficient oxygen and nutrients [90].

### 4.3 Immunosuppressive pathways of senescent macrophages in tumor immune evasion

Tumor cells evade immune surveillance and attack by modifying surface antigens and altering the TME, a phenomenon known as tumor immune evasion. Recent studies show that depletion of tissue-resident macrophages leads to reduced regulatory T (Tregs) cells and accumulation of CD8+ T cells, establishing an immunosuppressive TME that promotes tumor invasion and growth [108]. Senescent macrophages create an immunosuppressive milieu, weakening the ability of the immune system to recognize and eliminate tumors, thereby facilitating tumor immune evasion. Studies have shown that senescent macrophages contribute to the formation of an immunosuppressive TME (increased Tregs, decreased CD4+ and CD8+ T cells), whereas clearing senescent macrophages fosters a pro-immunogenic TME (reduced Tregs, increased CD4+ and CD8+ T cells) [90]. These findings show the pivotal role of senescent macrophages in generating an immunosuppressive TME, promoting early lung tumor growth, and offering new possibilities to overcome immune barriers to inhibit tumor growth.

### 4.4 Upregulation of immune checkpoint molecules by senescent macrophages

Senescent macrophages suppress immune cell functions by upregulating immune checkpoint molecules such as programmed death-ligand 1 (PD-L1) and CD47 [109]. PD-L1 binds PD-1 to T cells, inhibiting their immune activity. CD47 interacts with signal regulatory protein alpha (SIRPα) on macrophages, inhibiting phagocytosis and allowing tumor cells to evade immune clearance [110]. Studies have shown that senescent alveolar macrophages expressing p16 increase PD-L1 levels, fostering an immunosuppressive environment and increasing the senescent cell burden. Treatment with anti-PD-L1 antibodies that bind to Fcγ receptors on effector cells can clear PD-L1 and p16-positive cells [109]. Cancer cells hijack these inhibitory pathways by overexpressing checkpoint genes to evade immune surveillance. Phagocytosis checkpoints such as CD47, CD24, MHC-I, PD-L1, STC-1, and GD2 act as "don't eat me" signals or interfere with "eat me" signals, suppressing immune responses [111]. Therefore, inhibiting these anti-phagocytic signals or receptors represents a promising immunotherapeutic strategy, with antibodies and inhibitors targeting CD47 being investigated in various preclinical and clinical trials.

### 4.5 Senescent macrophages inhibit T cell activation

The inhibition of T cell activation by senescent macrophages is a key mechanism by which tumor immune evasion is promoted. Macrophages play a critical regulatory role in T cell activation, participating throughout the entire activation process [112]. Senescent macrophages exhibit reduced expression of MHC class II molecules and co-receptors CD80 and CD86 involved in antigen presentation [113], leading to diminished antigen-presenting capability. Additionally, the significant downregulation of Toll-like receptors (TLRs) on senescent macrophages further impairs their antigen-presenting function [114]. This hinders the binding of antigen-specific T cell receptors (TCRs) on T cells with antigen peptide-MHC complexes on antigen-presenting cells (APCs) and the interaction of multiple co-stimulatory molecules on the surfaces of T cells and APCs, both of which are essential for the first and second signals required for T cell activation.

The heterogeneous senescence-associated secretory phenotype (SASP) secreted by senescent macrophages directly inhibits T cell activation. Among these factors, TGF-β can suppress the proliferation and cytotoxicity of effector T cells and promote the generation of Treg cells, further inhibiting immune responses [115]. IL-10 has significant anti-inflammatory effects, inhibiting macrophage and dendritic cell functions, thereby reducing antigen presentation and indirectly lowering T cell activation [116]. Similarly, increased secretion of prostaglandin E2 (PGE2) by senescent macrophages with aging inhibits T cell function [117]. Studies have shown that depletion of senescent macrophages significantly increases the abundance of CD8+ T cells in tumor lesions [90], indicating that senescent macrophages can inhibit CD8+ T cell immune responses and affect the immune response to lung cancer [94, 118, 119].

## 5. Strategies targeting senescent macrophages in anti-tumor therapy

This section will focus on two innovative therapies targeting senescent macrophages: selective clearance or mitigation of senescent macrophages using senolytics and targeting the reduction of SASP factors secreted by these cells. These strategies have shown potential in preclinical and clinical trials, not only for enhancing the efficacy of existing immunotherapies but also providing novel therapeutic approaches for tumor patients with tumors, especially elderly patients.

### 5.1 Clearance or mitigation of senescent macrophages

Since the first report of senolytics (drugs that selectively eliminate senescent cells) in 2015, there has been increasing attention, with many senolytic drugs entering clinical trials [120, 121]. These drugs offer promising strategies for preventing or treating various human diseases and age-related conditions. First-generation senolytics, such as dasatinib and quercetin (D+Q), have been tested in preclinical models of several aging and disease conditions, including type 2 diabetes, as well as skeletal, cardiac, renal, hepatic, pulmonary, muscular, and neurological disorders [121]. Experiments have demonstrated that senescent alveolar macrophages can promote early lung tumorigenesis, and clearing these senescent macrophages can reduce the incidence of KRAS-driven lung cancer, laying a solid foundation for lung cancer treatment targeting senescent macrophages [26] [90]. Moreover, combining this approach with other immunotherapy methods has shown superior efficacy. For instance, clearing macrophages in aged mice using F4/80 antibodies significantly improved IL-2/anti-CD40 immunotherapy, enhancing T cell activity in the tumor microenvironment and achieving a tumor regression rate of up to 78%. This combined therapy showed better effects in aged mice than in young mice, offering new therapeutic hope for elderly patients [122]. Maintaining the normal immunosurveillance function of macrophages is an effective strategy to inhibit lung cancer progression. Studies have found that the IL-4-STAT6 pathway and Lamin A/C can inhibit macrophage senescence [123, 124]. Therefore, supplementing IL-4 and Lamin A/C provides new avenues for preventing macrophage senescence and improving lung cancer prognosis.

Additionally, senescent macrophages inhibit the activity of immune cells by upregulating immune checkpoint molecules, weakening their ability to kill tumor cells. Overexpression of immune checkpoint molecules such as PD-L1 and CD47 on senescent macrophages presents promising targets for anti-lung cancer therapy. Studies suggest that anti-PD-L1 antibodies might be a promising approach to activate immune responses, deplete senescent macrophages in chronic inflammatory tissues, and improve senescence-associated damage and systemic inflammation [109].

### 5.2 Targeting the reduction of SASP

Current research indicates that targeting the reduction of SASP secreted by senescent cells is a promising approach to cancer therapy, complementing classical chemotherapy methods to limit cancer incidence and tumor progression [87]. Various approaches have been taken to target SASP, which is regulated at multiple levels. SASP inhibitors can directly or indirectly attenuate SASP by inhibiting transcription factors such as nuclear factor (NF)-κB, JAK-STAT signaling pathways, serine/threonine protein kinase mTOR, mitochondrial complex 1 or 4-related targets, or other pathways involved in the induction and maintenance of SASP [125-127]. Studies have shown that signaling pathways like NF-κB and p38 MAPK are involved in the transcriptional regulation of SASP factors [128, 129]. Several p38 MAPK pathway inhibitors, such as SB203580 [128], CDD450, and CDD111 [87], have been reported to reduce certain components of SASP. mTOR has also been shown to regulate SASP expression in certain models [130], and the mTOR inhibitor rapamycin can reduce SASP produced by senescent cells [131, 132]. Some drugs targeting SASP components have been clinically applied. For instance, the IL-1 receptor antagonist anakinra, anti-IL-6 receptor antibody tocilizumab, and TNF-α inhibitors such as etanercept and infliximab are currently used to treat rheumatoid arthritis [133, 134]. Additionally, the IL-6 inhibitor sirukumab has shown promising results in phase III clinical trials for rheumatoid arthritis [135]. These drugs may help selectively block harmful SASP responses, thus altering the immunosuppressive tumor microenvironment created by senescent macrophages.

## 6. Challenges and Strategies for Clinical Translation

### 6.1 Enhancing Targeting Precision and Reducing Side Effects in Therapy

To effectively target senescent macrophages in lung cancer therapy while minimizing effects on normal immune function, the following key strategies should be considered:

(a) Identification of Specific Markers: One of the first steps in targeting senescent macrophages is identifying unique molecular markers that distinguish them from normal macrophages. High-throughput screening methods, followed by rigorous validation, can help pinpoint these markers. Such markers would serve as a foundation for developing targeted therapies that selectively affect senescent macrophages, reducing the risk of off-target effects on healthy immune cells.
(b) Precision Drug Delivery: The development of advanced drug delivery systems is crucial to ensuring the specific targeting of senescent macrophages. Nanotechnology-based drug carriers can be engineered to deliver therapeutic agents directly to the tumor microenvironment [136], where senescent macrophages are often concentrated. These precision carriers enable localized treatment, thereby minimizing systemic side effects and enhancing the effectiveness of the therapy by focusing on the tumor-associated macrophages.
(c) Combination Therapies: Combining senescent macrophage-targeting treatments with other immunotherapies, such as immune checkpoint inhibitors, could provide synergistic benefits. While targeting senescent macrophages might reprogram the tumor microenvironment, checkpoint inhibitors could further enhance the anti-tumor immune response [137]. This dual approach may not only increase therapeutic efficacy but also reduce the risk of resistance and the adverse effects associated with monotherapies [138].
(d) Comprehensive Monitoring: A rigorous monitoring system is essential to assess both the efficacy and safety of therapies targeting senescent macrophages. Tracking inflammatory markers, immune function indicators, and other biomarkers will help gauge the overall immune status of the patient during treatment. Early detection of any unwanted immune modulation or systemic side effects could guide timely adjustments in the therapy, ensuring that normal immune functions are not compromised.

By integrating these strategies, it is possible to enhance the specificity and safety of targeting senescent macrophages in lung cancer therapy. These approaches aim to increase therapeutic benefit while preserving the integrity of the patient’s overall immune system, ultimately improving both the safety and efficacy of anti-tumor treatments.

### 6.2 Translational Challenges from Experimental Research to Clinical Application

Although existing research has highlighted the role of senescent macrophages in lung cancer progression, these findings still have a considerable gap to clinical application. To target senescent macrophages for lung cancer therapy, foundational research needs to make breakthroughs in several areas, including in-depth studies of molecular mechanisms, biomarker identification and validation, the role of senescent macrophages in different lung cancer subtypes, and cross-species comparative analysis (Table 1). These studies will provide a solid theoretical foundation for clinical translation and help develop effective personalized treatment strategies. By strengthening these research areas, the clinical application of senescent macrophage-targeted therapies can be accelerated, improving lung cancer treatment outcomes and patient survival quality.

**Table 1:** Key Research Areas and Measures for Translating Senescent Macrophage-Targeted Therapy in Lung Cancer into Clinical Application.

Research Area	Specific Measures to Strengthen
Molecular Mechanisms of Senescent Macrophages	In-depth investigation of the mechanisms by which senescent macrophages contribute to lung cancer progression, particularly their role in immune evasion, tumor metastasis, and promotion of tumor growth.
Biomarker Screening and Validation	Screen and validate senescent macrophage-specific biomarkers to ensure their universality and clinical relevance across different lung cancer subtypes.
Role in Different Lung Cancer Subtypes	Study the functional differences of senescent macrophages in different lung cancer subtypes to provide insights for personalized treatment strategies.
Dynamic Monitoring of Senescent Macrophages	Track the functional changes of senescent macrophages at different tumor stages to reveal their roles in tumor progression and metastasis.
Cross-Species Consistency	Compare the characteristics of senescent macrophages in mouse models and human tumor microenvironments to enhance the translational relevance of research findings [139].
Immune Regulation Mechanisms	Explore the interactions between senescent macrophages and other immune cells (such as T cells and NK cells) to understand their role in immune escape mechanisms in lung cancer.
Clinical Application of Biomarkers	Develop senescent macrophage-related biomarkers for early diagnosis, prognosis assessment, and monitoring treatment efficacy in lung cancer.
Combination Strategies with Immunotherapy	Investigate the combination of senescent macrophage-targeted therapies with existing immunotherapeutic approaches (e.g., immune checkpoint inhibitors) to enhance therapeutic outcomes.

## 6. Conclusion

In the lung cancer microenvironment, the role of senescent macrophages presents a novel therapeutic target. Strategies targeting these cells may enhance lung cancer treatment, particularly as immunotherapy becomes a standard therapeutic approach for lung cancer. Understanding the interactions between senescent macrophages and lung cancer cells, as well as the mechanisms by which they contribute to tumor immune evasion, is not only scientifically innovative but also clinically significant. This study reveals the substantial impact of senescent macrophages in the lung cancer microenvironment, where they influence tumor growth and immune evasion through various mechanisms, such as the secretion of suppressive cytokines and modulation of immune checkpoint molecules. Therapeutic approaches targeting these macrophages hold great potential and may emerge as key strategies in lung cancer immunotherapy.

However, translating these findings into clinical application presents multiple challenges. First, a deeper understanding of the molecular mechanisms underlying senescent macrophages is crucial for developing more precise therapeutic strategies. Furthermore, further clinical research is essential to validate the effectiveness and safety of these approaches. Only through interdisciplinary collaboration and sustained research efforts can we facilitate the translation of basic research into clinical practice, offering more effective treatment options for lung cancer patients.
